# Comprehensive Multi‐Neuron Imaging During Aging and Degeneration in *C. elegans*

**DOI:** 10.1002/alz.089961

**Published:** 2025-01-03

**Authors:** Dilip Yadav, Gregory Wirak, Christopher Connor, Chritopher Gabel

**Affiliations:** ^1^ Chobanian & Avedisian School of Medicine, Boston University, Boston, MA USA; ^2^ Brigham & Women’s Hospital, Harvard Medical School, Boston, MA USA

## Abstract

**Background:**

Despite the prevalence of Alzheimer’s Disease in the aged human population and advancements in our understanding of the disease at the molecular level, we still lack a fundamental understanding of how cognitive dysfunction stems from alterations in cellular neuron dynamics and how it relates to the normal aging process. To address this gap in knowledge, we measured the breakdown of nervous system function with cellular resolution during normal aging and in an Alzheimer’s Disease model.

**Method:**

Employing comprehensive multi‐neuron florescence microscopy in the nematode worm, *C. elegans*, a simple yet powerful model animal, we can measure neuron activity across the majority of the animal’s nervous system with single cell resolution. To measure how system‐state dynamics and neuronal connectivity breakdown during normal aging and in the disease model, imaging was performed throughout the animal’s lifespan in wild‐type as well as a transgenic strain in which human amyloid‐β (Aβ) peptide (1‐42) is pan‐neuronally expressed.

**Results:**

During normal aging we measure a breakdown in system‐state dynamics that accompany shifts in activity to higher frequencies and a global loss of inhibitory signaling. In Aβ expressing animals this process is accelerated and includes loss of both inhibitory and excitatory signaling.

**Conclusion:**

Our work is defining the functional degradation of a complete nervous system from single neurons to system‐wide dynamics during normal aging and in the disease model. In doing so we are revealing the specific changes in neuron activity at the cellular level that cause the breakdown of global nervous system dynamics. These results will enable a knowledge‐based approach to novel neurotherapeutic strategies in the treatment and prevention of dementia.

